# New advances in understanding the mechanisms and treatment challenges of ALK-targeted therapy resistance in lung cancer

**DOI:** 10.20517/cdr.2025.122

**Published:** 2025-08-25

**Authors:** Mengle Long, Shixuan Peng, Qingyang Wen, Zhijian Yin, Xinwen Zhang, Haoyu Tan, Yun Xu, Yongjun Wu

**Affiliations:** ^1^Department of Oncology, Graduate Collaborative Training Base of The First People’s Hospital of Xiangtan City, Hengyang Medical School, University of South China, Hengyang 421001, Hunan, China.; ^2^Department of Oncology, The First People’s Hospital of Xiangtan City, Xiangtan 411101, Hunan, China.; ^3^Department of Oncology, Graduate Collaborative Training Base of Xiangtan Center Hospital, Hengyang Medical School, University of South China, Hengyang 421001, Hunan, China.; ^4^Department of Pathology, Xiangtan Central Hospital, Xiangtan 411100, Hunan, China.; ^5^Department of Rehabilitation Medicine, The First People’s Hospital of Xiangtan City, Xiangtan 411101, Hunan, China.; ^6^Department of Pathology, The Affiliated Hospital of Hunan University, Xiangtan 411100, Hunan, China.; ^#^Authors contributed equally.

**Keywords:** Anaplastic lymphoma kinase (ALK), drug resistance mechanisms, mutations, small cell transformation, immune microenvironment, targeted therapy

## Abstract

Despite the development of various effective anaplastic lymphoma kinase tyrosine kinase inhibitors (ALK-TKIs), therapeutic resistance remains a major challenge. Both on-target and off-target mechanisms have been identified as key contributors to resistance. With the popularization of genetic testing and the development of precision therapies, the prognosis and survival of patients with ALK-positive non-small cell lung cancer (NSCLC) have improved. However, even with second- and third-generation ALK-TKIs, overcoming resistance remains difficult. Resistance frequently arises during approved treatments, underscoring the need for further research to elucidate the molecular events and resistance mechanisms associated with ALK-positive lung cancer. The discovery of anaplastic lymphoma kinase (ALK) rearrangement as an actionable oncogenic driver in NSCLC has established a biomarker-driven treatment paradigm for advanced disease. This article summarizes current knowledge of the mechanisms of resistance to ALK-targeted therapy in lung cancer, including both primary and acquired mechanisms, treatment strategies following resistance, recent therapeutic advances, and the impact of the immune system and tumor microenvironment. A deeper understanding of ALK-targeted therapy resistance is critical for developing new treatment strategies and may provide important insights to guide the diagnosis, treatment, and management of patients with resistant ALK^+^ lung cancer.

## INTRODUCTION

Lung cancer remains the leading cause of cancer-related mortality worldwide, with a persistently low five-year overall survival (OS) rate^[1]^. Non-small cell lung cancer (NSCLC) accounts for about 85% of all lung cancer cases. Targeted therapy has marked a significant advancement in lung cancer treatment and has successfully improved the five-year survival rate of patients with advanced NSCLC^[2]^. However, the emergence of drug resistance during targeted therapy is common, with resistance to anaplastic lymphoma kinase (ALK) inhibitors being one of the most frequent challenges^[3]^. Approximately two-thirds of NSCLC patients harbor gene mutations, and the incidence of ALK rearrangements in NSCLC is 3%-7%^[4]^. ALK can fuse or rearrange with multiple partner genes, among which echinoderm microtubule-associated protein-like 4-ALK (EML4-ALK) is the most common fusion type^[5-7]^.

ALK is regarded as a “diamond target”; however, multiple drug resistance pathways emerge during treatment with ALK tyrosine kinase inhibitors (ALK-TKIs). Even with second- and third-generation ALK-TKIs, overcoming resistance remains a major clinical obstacle^[8]^. The discovery of ALK rearrangements as operable carcinogenic drivers in NSCLC has fostered a biomarker-oriented treatment paradigm for advanced disease. To date, many ALK-TKIs have been developed and shown to be effective in NSCLC with epidermal growth factor receptor (EGFR) mutations or ALK rearrangements, leading to the approval of highly potent third-generation TKIs such as Osimertinib and Lorlatinib^[9,10]^ [Table 1]. Although these drugs demonstrate remarkable efficacy, resistance remains a major unresolved issue. In patients receiving advanced treatment with Osimertinib or Lorlatinib, resistance mechanisms can be broadly categorized into two types: “on-target” resistance, primarily caused by secondary mutations in the EGFR or ALK kinase domains, and “off-target” resistance, involving alterations in non-target kinases such as bypass pathway activation or phenotypic transformation^[29,30]^.

**Table 1:** ALK inhibitors and associated mutations in FDA-approved drugs or ongoing clinical trials

**Drug**	**Target of mutation**	**Sensitive mutations**	**ALK resistance mutations**	**Regulatory approval**	**Phase of testing**	**Ref.**
**1st generation**						
Crizotinib (PF-02341066)	ALK rearrangement; ROS1 rearrangement; c-MET	L1198F, E1210K	L1196M, G1269A/S, S1206Y, V1180L, G1202R, C1156Y, I1151Tins, F1174C/L, L1152R/P, L1198P, I1171N/T/S, D1203N	Approved for first-line and subsequent treatment (previously treated patients)	Phase III, completed	[11-15]
**2nd generation**						
Alectinib (RO/CH5424802)	ALK rearrangement; RET; GAK; LTK	C1156Y/T, L1198F, G1269A/S, S1206Y, L1152P/R, F1174C/L/V, I1151Tins	L1196M, V1180L, G1202R, I1171N/T/S, G1269A, S1206Y, F1174L	Approved for first-line and post-crizotinib use	Phase III	[16-18]
Ceritinib (LDK378)	ALK rearrangement; ROS1; IGF1R	C1156Y/T, I1171 N/T/S, L1196M, G1269A/S, S1206Y	G1202R, C1156Y, I1151Tins, F1174C/L, L1152R/P	Approved for crizotinib-pretreated patients	Phase III	[19-21]
Brigatinib (AP26113)	ALK rearrangement; ROS1; EGFR	C1156Y/T, I1171 N/T/S, L1196M, G1269A, S1206Y, L1198F, L1152R, F1174C/L/V, I1151Tins	S1206C, E1210K, G1202R, E1210K, D1203N	Breakthrough therapy designation for crizotinib-pretreated patients	Phase III	[20-23]
**3rd generation**						
Lorlatinib (PF-06463922)	ALK rearrangement; ROS1 rearrangement	C1156Y/T, I1171N/T/S, L1196M, G1202R, G1269A/S	L1198F, L1256F, G1202R, S1206C/Y, I1171N/T/S, C1156Y, L1196M	EMA: not yet approved; FDA: approval pending	Phase III	[24-26]
Ensartinib (X-396)	MET; ROS1; ALK	T1151M, G1269A, L1196M, G1202R	L1198F, G1269A, E1210K, G1202R	EMA: not yet approved; FDA: not yet approved	Phase III	[20,27,28]

ALK: Anaplastic lymphoma kinase; ROS1: ROS proto-oncogene 1; EGFR: epidermal growth factor receptor; MET: mesenchymal-epithelial transition.

The clinical application of second-generation ALK-TKIs such as alectinib has substantially improved prognosis and survival compared with the chemotherapy era for patients with ALK-positive NSCLC^[31]^. With the continued progress in precision oncology, molecular testing has become widely adopted in NSCLC management. Since the first report of EML4-ALK rearrangements in NSCLC, numerous additional ALK fusion partners have been identified^[32,33]^. Consequently, increasing attention has been directed toward the management of ALK-targeted drug resistance in lung cancer. To provide a clearer understanding of its pathogenesis, the role of the immune system and tumor microenvironment, as well as potential therapeutic strategies, this review summarizes current knowledge of the mechanisms and treatment challenges associated with ALK-targeted drug resistance in lung cancer.

## MECHANISM OF ALK-TARGETED DRUG RESISTANCE IN LUNG CANCER

### Point mutations in the ALK kinase domain

**Figure 1:**
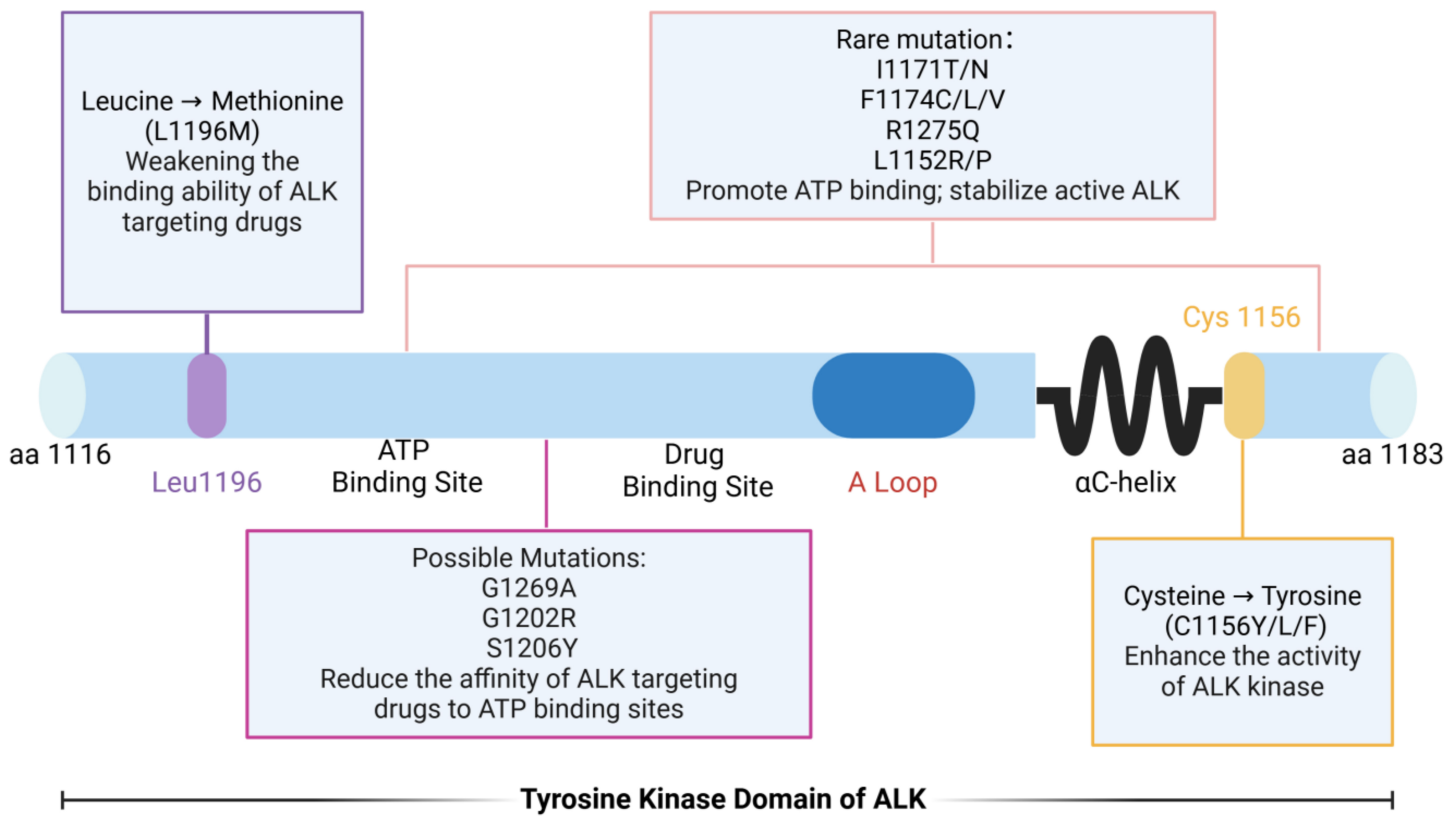
Point mutations within the ALK tyrosine kinase domain and their functional impact. Schematic representation of the ALK tyrosine kinase domain (residues 1116-1183), illustrating the location and functional consequences of representative resistance-associated mutations. The gatekeeper mutation L1196M impairs drug binding. Mutations such as G1202R, S1206Y, and G1269A reduce inhibitor affinity at the ATP-binding pocket. Variants including I1171T/N, F1174C/L/V, R1275Q, and L1152R/P increase ATP binding and stabilize the active kinase conformation. Substitutions at C1156 (C1156Y/L/F) enhance ALK kinase activity, further promoting resistance. Created in BioRender. Long, M. (2025). https://BioRender.com/4hhix6x. ALK: Anaplastic lymphoma kinase; ATP: adenosine triphosphate.

Point mutations in the *ALK* gene represent a primary mechanism underlying resistance to ALK-targeted therapies^[34]^. To date, numerous mutations have been identified within the ALK kinase domain. The most common include: L1196Mlocated in the first peak region of the kinase domain, this “gatekeeper” mutation reduces the binding affinity of ALK inhibitors, thereby conferringdrug resistance^[35,36]^; G1202R - found in the second peak region of the kinase domain, this mutation increases ATP-binding affinity, which diminishes the effectiveness of drugs^[37-39]^. S1206Y - also located in the second peak region, this substitution decreases inhibitor binding and promotes resistance; C1156Y/L/F - these variants enhance ALK kinase activity, contributing to resistance against targeted therapy. In addition, several less frequent mutations, such as I1171T/N, F1174C/L/V, R1275Q, and L1152R, also drive therapeutic resistance. Among these, L1196M and G1269A are the most prevalent. Both occur near the ATP-binding pocket and the drug-binding site, directly impairing inhibitor binding^[40,41]^ [Figure 1]. Collectively, these kinase domain mutations disrupt inhibitor binding or enhance kinase activity, representing one of the primary mechanisms of targeted therapy failure. Therefore, comprehensive profiling of such mutations and the development of next-generation inhibitors are critical to extending the therapeutic efficacy of ALK-directed treatment.

### Bypass signaling pathway activation and resistance to ALK-targeted therapy

**Figure 2:**
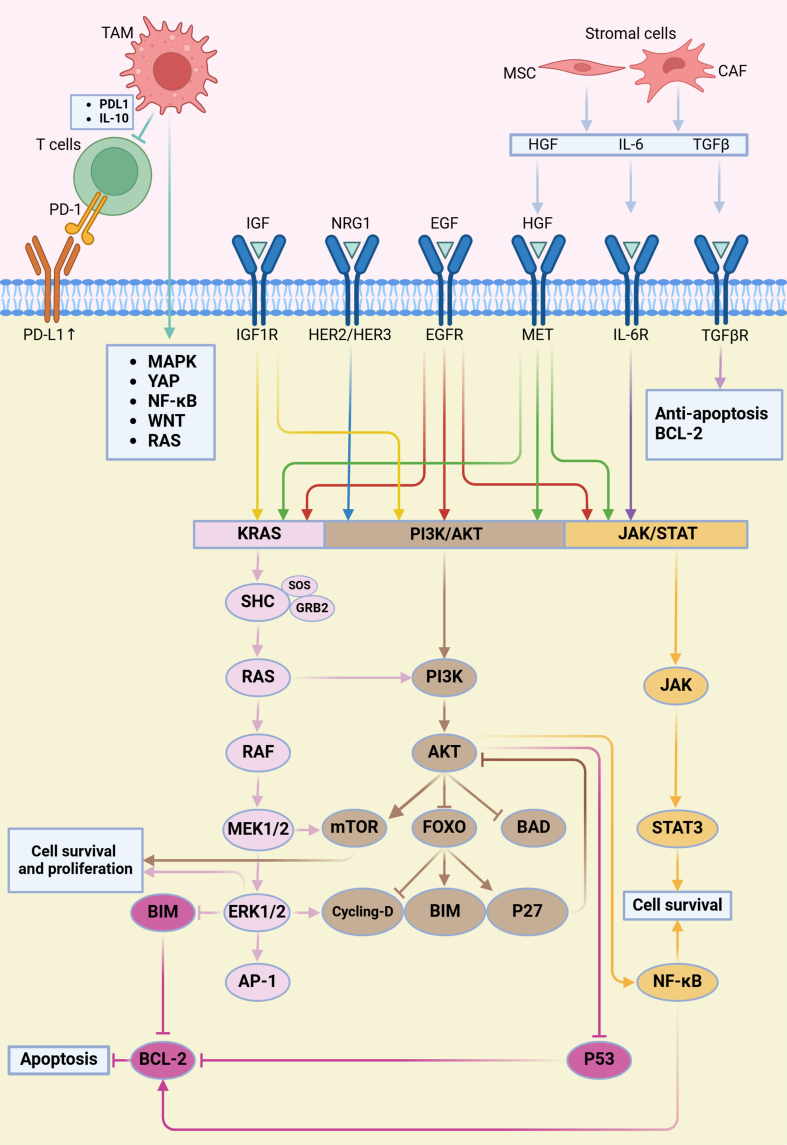
ALK-TKI resistance mediated by the tumor microenvironment and bypass signaling activation. This figure illustrates how the tumor microenvironment promotes resistance to ALK-TKIs through activation of bypass signaling pathways. Stromal components such as CAFs and MSCs secrete cytokines and growth factors including IL-6, TGFβ, and HGF. Together with ligands such as IGF, NRG1, and EGF, these molecules activate corresponding receptors on tumor cells. Receptor activation triggers downstream signaling pathways including RAS–RAF–MEK–ERK, PI3K–AKT, and JAK–STAT, ultimately enhancing cell survival and inhibiting apoptosis through mediators such as BCL-2 and NF-κB. This process reduces the effectiveness of ALK inhibitors. Additionally, TAMs contribute to immune evasion by secreting IL-10 and inducing PD-L1 expression on tumor cells, thereby suppressing T cell-mediated antitumor immunity. Both TAMs and T cells further modulate oncogenic signaling by releasing factors that activate the MAPK, YAP1, NF-κB, WNT, and RAS pathways. Created in BioRender. Long, M. (2025). https://BioRender.com/4cy0tmf. ALK-TKI: Anaplastic lymphoma kinase tyrosine kinase inhibitor; CAFs: cancer-associated fibroblasts; MSCs: mesenchymal stem cells; PI3K: phosphatidylinositol 3-kinase; JAK: Janus kinase; STAT: signal transducer and activator of transcription; NF-κB: nuclear factor-κB; TAMs: tumor-associated macrophages; PD-L1: programmed death ligand 1; YAP1: Hippo-Yes-associated protein 1.

ALK is a receptor tyrosine kinase whose aberrant activation in lung cancer promotes cell proliferation, survival, and metastasis. ALK-targeted therapies, including agents such as Crizotinib and Alectinib, have been extensively employed in the treatment of ALK-positive NSCLC^[42]^ [Table 1]. However, due to the complexity of intracellular signaling networks, tumor cells frequently develop resistance, leading to the activation of alternative pathways that bypass ALK inhibition. The main bypass signaling pathways implicated in ALK-targeted therapy resistance are outlined below [Figure 2].

#### EGFR-mediated bypass activation

The EGFR is a receptor tyrosine kinase that interacts closely with ALK signaling. In ALK-positive NSCLC, EGFR mutations and overexpression have been associated with resistance to ALK inhibitors^[43]^. EGFR activation stimulates downstream signaling molecules independently of ALK, thereby promoting resistance to therapy^[44]^. Among bypass mechanisms, EGFR activation is one of the most frequent, particularly after treatment with first- and second-generation ALK inhibitors such as Crizotinib, Ceritinib, and Alectinib. At the molecular level, increased EGFR autophosphorylation and upregulation of EGFR ligands reactivate downstream cascades such as the phosphatidylinositol 3-kinase (PI3K)/AKT and MAPK/ERK pathways, enabling tumor cells to reduce dependence on ALK signaling^[41]^. Notably, heparin-binding EGF-like growth factor (HB-EGF) has been reported to induce crizotinib resistance by activating EGFR and stimulating the ERK1/2 and AKT pathways^[45]^. These findings highlight the critical role of EGFR-mediated bypass signaling in limiting the long-term efficacy of ALK inhibitors.

#### KRAS-mediated bypass activation

Kirsten rat sarcoma viral oncogene homolog (KRAS) is a small GTPase that regulates cell growth and survival. KRAS mutations contribute to ALK inhibitor resistance in ALK-positive NSCLC by activating downstream signaling pathways, including MAPK/ERK and PI3K/AKT. These mutations are linked to poor prognosis and higher recurrence risk^[46,47]^. In addition, recent studies have shown that in BRAF-mutant melanoma, aberrant ALK activation can restore MAPK pathway signaling through bypass mechanisms, conferring resistance to BRAF inhibitors (BRAFi)^[48]^. Overexpression or fusion of ALK can reinstate ERK phosphorylation and sustain tumor proliferation, suggesting that ALK inhibitors may help overcome BRAFi resistance. Conversely, although direct evidence in NSCLC is limited, MAPK pathway reactivation - including BRAF and MEK/ERK signaling - has been observed in ALK inhibitor resistance models, potentially reducing the efficacy of agents such as Ceritinib and Alectinib^[49,50]^. Collectively, BRAF and KRAS mutations appear to converge with ALK signaling on shared downstream pathways, driving resistance phenotypes. Given its clinical significance, aberrant KRAS pathway activation in NSCLC has become an active focus of therapeutic research^[51]^.

#### PI3K/AKT-mediated bypass activation

The PI3K/AKT pathway is essential for cell survival and proliferation^[52]^. Hyperactivation of this pathway enhances proliferation, survival, and metastasis in lung cancer, and has been implicated in resistance to ALK inhibitors^[53,54]^. In ALK-TKI resistance, PI3K/AKT activation often occurs independently of ALK signaling, supporting tumor growth by suppressing pro-apoptotic proteins and amplifying anti-apoptotic signals. Clinical and preclinical studies have demonstrated that the PI3K/AKT pathway is reactivated in ALK-positive NSCLC after Crizotinib and other TKI treatments, often through bypass activation involving EGFR or IGF-1R. Notably, secondary EGFR mutations that trigger PI3K/AKT reactivation have been linked to crizotinib resistance and diminished therapeutic responses^[55]^. These findings underscore the importance of simultaneously targeting ALK and key downstream effectors such as PI3K/AKT to overcome acquired resistance in ALK-positive NSCLC.

#### JAK/STAT-mediated bypass activation

The Janus kinase/signal transducer and activator of transcription (JAK/STAT) pathway regulates cell growth and differentiation. In ALK-positive NSCLC, aberrant activation of the JAK/STAT pathway has been correlated with resistance to ALK inhibitors^[56]^. This signaling cascade promotes tumor proliferation by downregulating cell cycle inhibitors and upregulating cell cycle-promoting molecules^[57]^.

Beyond these well-established pathways, others - including the Wnt/β-catenin and NF-κB pathways - may also participate in bypass-mediated resistance to ALK-targeted therapy^[58,59]^. Their aberrant activation further compromises the effectiveness of ALK inhibitors. Consequently, overcoming bypass resistance requires a deeper mechanistic understanding of signaling networks in lung cancer, which will inform the development of novel therapeutic strategies and improve the long-term efficacy of ALK-targeted treatments.

### Small cell transformation in the progression of ALK-targeted drug resistance in lung cancer

**Figure 3:**
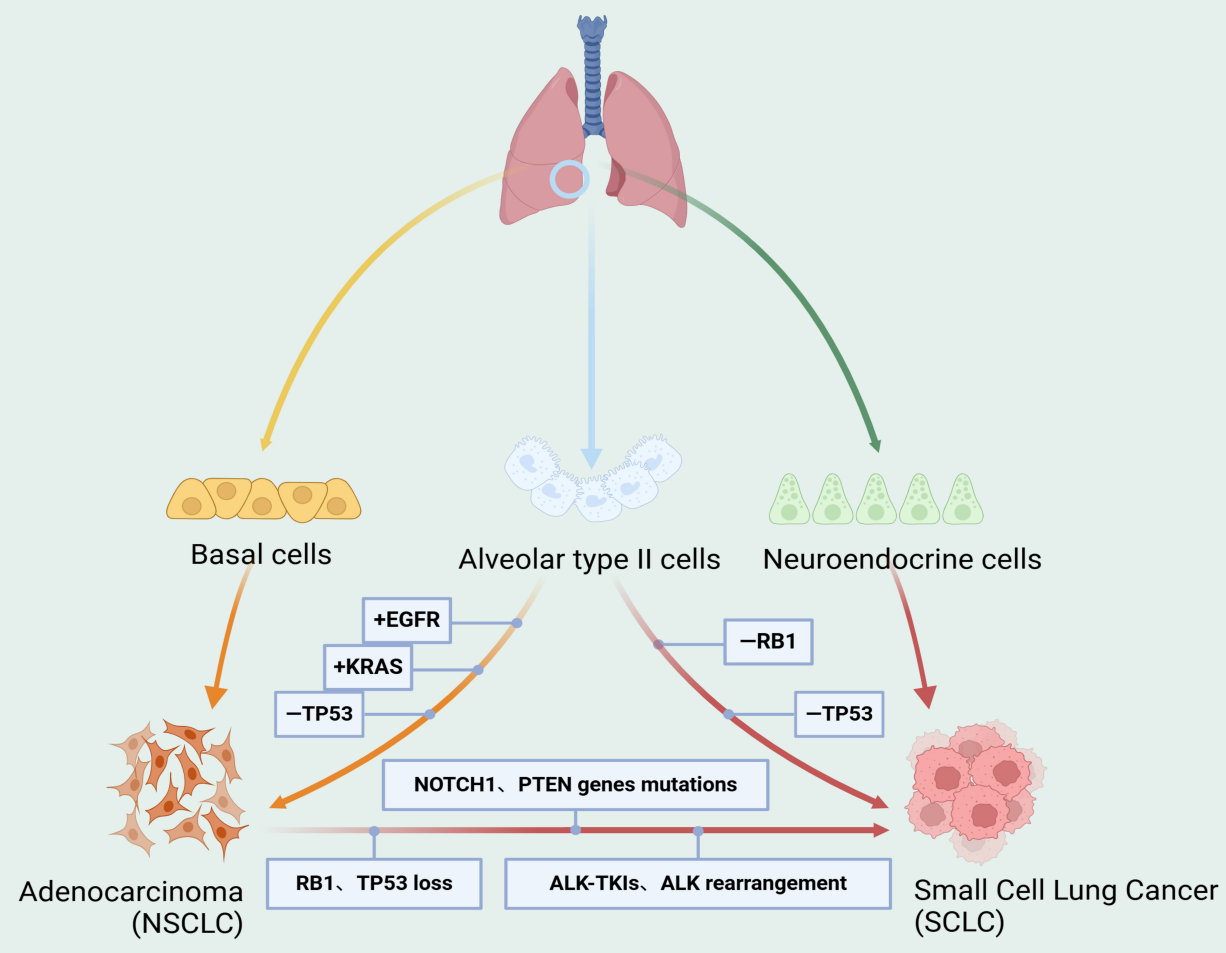
Mechanisms of small cell transformation in the progression of ALK-targeted resistance in lung cancer. SCLC can originate from neuroendocrine cells, while adenocarcinoma typically arises from basal cells. Alveolar type II cells may serve as a potential origin for both SCLC and adenocarcinoma. During SCLC transformation, multiple genetic alterations and signaling pathway activations are involved, including *RB1* and *TP53* loss, mutations in genes such as *NOTCH1* and *PTEN*, ALK-TKI resistance, and ALK rearrangements. Created in BioRender. Long, M. (2025). https://BioRender.com/ogqzb3a. SCLC: Small cell lung cancer; ALK-TKI: anaplastic lymphoma kinase tyrosine kinase inhibitor.

Small cell lung cancer (SCLC) is a highly malignant neuroendocrine subtype of lung cancer with a poor prognosis. It is characterized by aggressive behavior, rapid tumor growth, and widespread metastases^[60]^. Transformation into SCLC represents a resistance mechanism through which NSCLC can evade immunotherapy, chemotherapy, and targeted therapy. In a subset of patients with ALK-positive NSCLC, histologic transformation to SCLC may occur, resulting in substantial resistance to ALK-targeted therapy. The 5-year survival rate for SCLC remains extremely low, at approximately 5%-10%, compared with NSCLC, which generally exhibits slower growth and delayed invasion or metastasis^[61]^. Transformed SCLC is considered a distinct phenotype from de novo SCLC, although the two share certain molecular, histopathological, and clinical features, as well as similar patterns of drug responsiveness. Nonetheless, de novo SCLC and transformed SCLC differ in their underlying pathogenesis and tumor microenvironment [Figure 3]. In ALK-positive NSCLC, SCLC transformation typically emerges after exposure to ALK inhibitors, signifying advanced drug resistance. This process may be associated with reduced expression of the ALK fusion gene, dysregulation of cell proliferation signaling pathways, and altered transcription factor activity^[62]^.

For NSCLC patients who undergo SCLC transformation, timely confirmation of diagnosis and initiation of individualized treatment are critical. To overcome this resistance mechanism, further investigation into the molecular basis of transformation and the development of corresponding treatment strategies are urgently needed. Currently, no clear or standardized treatment strategy exists for ALK-positive NSCLC patients who develop SCLC transformation. Early studies suggest that traditional SCLC treatments, such as chemotherapy and radiotherapy, may provide some benefit in this setting^[63,64]^. In addition, novel therapeutic drugs targeting SCLC are under development, though no consensus standard of care is available for patients who experience transformation after ALK-TKI therapy. Re-biopsy at the time of acquired resistance has been recommended to guide alternative treatment selection^[65]^. For instance, Cisplatin combined with Irinotecan (the standard SCLC regimen in Japan) has achieved sustained partial remission of primary tumors as well as partial remission at metastatic sites^[66]^. These findings underscore the need to promptly adjust and individualize treatment plans for patients with SCLC transformation to maximize therapeutic outcomes.

Preliminary studies have reported clinical outcomes in patients with ALK-positive NSCLC who develop SCLC transformation. A systematic review analyzing 17 cases of lung adenocarcinoma and 16 cases treated with first- or second-generation TKIs found that patients had a median OS of 6 months following SCLC transformation, whereas those treated solely with Osimertinib had a median OS of just 2 months^[67]^. Another study of 58 patients with NSCLC reported a median time to transformation of 17.8 months, a median OS of 31.5 months from initial diagnosis, and a median survival of 10.9 months following transformation^[68]^. Biopsy remains the most reliable method for confirming SCLC transformation. Conventional SCLC regimens, including chemotherapy and radiotherapy, may offer limited efficacy in these cases^[69]^. However, several challenges persist. On one hand, conventional treatments are often associated with substantial toxicity, which may significantly affect quality of life. On the other hand, novel therapies for SCLC remain in the early stages of development, with their long-term safety and efficacy yet to be established^[70,71]^. Therefore, when managing ALK-positive NSCLC patients who experience SCLC transformation, it is essential to consider the patient’s overall condition, prior treatment history, and quality of life in tailoring an appropriate therapeutic plan. Meanwhile, further research into the mechanisms of SCLC transformation is needed to identify novel therapeutic targets and strategies, ultimately expanding treatment options and improving outcomes for these patients.

## THE RELATIONSHIP BETWEEN ALK-TARGETED THERAPY AND THE IMMUNE MICROENVIRONMENT

Although immune checkpoint inhibitors (ICIs) represent a key treatment option for advanced NSCLC, patients with ALK-rearranged NSCLC generally fail to derive significant clinical benefit^[72,73]^. This lack of response may be attributed to the unique features of the TME^[74]^. Research indicates that the immune microenvironment plays a critical role in the development of resistance to ALK-targeted therapy. Initially, ALK inhibitors can enhance immune cell infiltration and activation, promoting cancer cell apoptosis. However, prolonged treatment may gradually alter the TME, resulting in reduced immune cell infiltration and activation, allowing lung cancer cells to escape immune surveillance and develop therapeutic resistance^[75]^. Moreover, ALK-targeted therapy can modulate the expression and activity of immune checkpoint molecules, including CTLA-4, programmed death ligand 1 (PD-L1), and PD-1, which suppress immune cell function and further facilitate immune escape. To date, no survival analyses have been conducted based on PD-L1 expression in ALK-positive patients receiving TKI therapy. Instead, existing studies have primarily assessed the immune landscape using markers such as PD-L1, PD-1, CD3, and CD8^[76-78]^. Therefore, therapeutic strategies aimed at enhancing immune cell activity - such as combining ICIs with ALK-targeted agents - may help overcome resistance and improve outcomes in lung cancer patients.

The advent of ALK-targeted therapy has significantly improved survival rates in lung cancer. Nevertheless, during treatment, patients often develop local and systemic immune dysregulation, especially when resistance to ALK inhibitors emerges. In resistant cases, both the number and composition of immune cells within the TME - including B cells, T cells, and natural killer (NK) cells - are altered. These immune cells can interact with tumor cells, driving further progression and metastasis^[79]^. Importantly, ALK-targeted therapies can reshape the immune microenvironment in ALK fusion-positive NSCLC. Such therapies may increase immune cell infiltration while suppressing the expression of immunosuppressive molecules, thereby enhancing the immune system’s ability to recognize and eliminate tumor cells. This effect could improve patient responsiveness to immunotherapy^[80]^. Taken together, understanding the complex interactions between ALK-targeted therapy and the immune microenvironment is crucial for developing more effective treatment approaches and improving clinical outcomes in patients with lung cancer.

## ALK-TARGETED THERAPY RESISTANCE IN LUNG CANCER AND IMMUNE ESCAPE RELATED TO IMMUNE CHECKPOINTS

Resistance to ALK-targeted therapy in lung cancer is also related to immune escape mechanisms involving immune checkpoints. Studies have shown that PD-L1 expression is significantly elevated in patients with ALK-targeted therapy resistance, which may enable tumor cells to evade immune detection and elimination^[81,82]^. Consequently, ICIs may enhance treatment outcomes in patients with ALK-resistant lung cancer. Immune escape contributes to the development of resistance to ALK-targeted therapies by allowing tumor cells to avoid recognition and clearance by the immune system through multiple mechanisms^[83]^. In lung cancers resistant to ALK-targeted drugs, tumor cells may achieve immune escape via several pathways: First, they may downregulate the expression of tumor antigens, reducing immune system recognition and attack^[84]^. Second, they may activate immunosuppressive pathways, including the PD-L1/PD-1 and CTLA-4 axes, thereby suppressing immune cell activation. Additionally, tumor cells may secrete immunosuppressive factors such as TGF-β and IL-10, which induce immune tolerance and further prevent immune-mediated attack. Upregulation of immune checkpoint molecules such as PD-L1 represents a key mechanism by which tumor cells evade immune surveillance following resistance to ALK-targeted therapy.

Current therapeutic strategies to overcome immune escape in ALK-targeted therapy-resistant lung cancer mainly involve immunotherapy and combination therapy. Immunotherapy aims to inhibit immunosuppressive pathways or enhance immune cell activation to strengthen the immune system’s ability to attack tumor cells. Combination therapy, on the other hand, employs multiple treatment modalities, such as targeted therapy, radiotherapy, chemotherapy, and immunotherapy, simultaneously to attack tumor cells from different angles, thereby improving treatment efficacy and tolerability^[85]^. Addressing immune escape is a crucial aspect of managing ALK-targeted therapy resistance, and treatment strategies must consider tumor characteristics, patient condition, therapeutic efficacy, and safety.

## METHODS FOR TREATING ALK-TARGETED THERAPY-RESISTANT LUNG CANCER

Lung cancers harboring the ALK fusion gene can often be controlled with ALK-targeted inhibitors, which help prevent disease progression. However, long-term use of these drugs can lead to resistance, posing a significant challenge in clinical management. Developing new strategies to treat ALK-targeted therapy-resistant lung cancer is therefore crucial^[86,87]^. Currently, several approaches have been explored to overcome ALK resistance [Table 2], including the following:

**Table 2:** Summary of alternative strategies for the treatment of targeted drug resistance in ALK

**Drug regimen**	**Study phase**	**Results**	**Clinical trials/ref.**
KIF5B-RET inhibitor (LOXO-292)	I	Ongoing	NCT03157128
Immunotherapy (PD-L1 inhibitors)	Preclinical/clinical	Upregulation of PD-L1. Demonstrated marked antitumor efficacy compared with monotherapy in preclinical ALK-positive NSCLC models. In clinical trials, combinations showed some activity but with severe adverse events	[85,88-91]
Targeting drug-tolerant Persister cells	IV	Local consolidative approaches used to eliminate persister cells in areas of residual disease	NCT02314364 [92]
**Multitarget therapy**			
Alectinib + Bevacizumab (anti-VEGF mAb)	I/II	Upfront ALK and VEGFR inhibition re-sensitized ALK-TKI-resistant ALK-positive NSCLC cell lines. Lorlatinib plus bevacizumab achieved disease regression	NCT02521051 [93,94]
Ceritinib + Everolimus (mTOR inhibitor)	I/Ib	Combination of ALK and mTOR inhibition significantly reduced the proliferation of ALK-TKI-resistant cell lines	NCT02321501 [95-98]
Ceritinib + Trametinib (MEK inhibitor)	Preclinical/clinical	Enhanced the magnitude and duration of initial drug response in untreated ALK-positive NSCLC cell lines and overcame resistance in ALK-TKI-resistant lines	NCT03087448 NCT03202940 [99-104]
Crizotinib + Dacomitinib (HER2 inhibitor)	I	Excessive toxicity	NCT01121575 [96]
Lorlatinib + Crizotinib	I/II	Unknown	NCT04292119 [105]
Lorlatinib + TNO155	I/II	Unknown	NCT04292119 [106]
EGFR inhibitors	Preclinical	Improved therapeutic efficacy compared with ALK-TKI monotherapy	[107-111]
**New ALK-targeting drugs**			
TPX-0131	I	Terminated (adverse change in risk-benefit ratio)	NCT04849273
NVL-655 (Neladalkib)	I/II	Ongoing	NCT05384626
WX-0593	II	Ongoing (recruiting)	NCT04641754
TQ-B3139	II	Unknown	NCT04056572
**Combination therapy**			
Chemotherapy (Irinotecan) + Alectinib	/	Partial response in primary lesion and metastases	[65]
Immunotherapy (Nivolumab) + Ceritinib	Ib	Demonstrated activity	[91]
Ipilimumab (CTLA-4 inhibitor) + Nivolumab (PD-1 inhibitor)	/	Improved response rates in advanced NSCLC	[106]

ALK: Anaplastic lymphoma kinase; PD-L1: programmed death ligand 1; NSCLC: non-small cell lung cancer; TKI: tyrosine kinase inhibitor; EGFR: epidermal growth factor receptor.

### KIF5B-RET targeted therapy

KIF5B-RET fusion genes are more frequently observed in ALK-negative lung cancers, accounting for approximately 5%-10% of cases. This fusion gene leads to a unique tumor biological phenotype that is highly sensitive to RET-targeted drugs, offering a promising therapeutic option. Several RET inhibitors have demonstrated encouraging efficacy in clinical trials. Therefore, KIF5B-RET targeted therapy presents a potential strategy for patients with ALK-resistant lung cancer^[112,113]^. RET-targeted drugs mainly include polypeptide amides, pyrrolothiazoles, triazoles, and related compounds. These agents act by directly or indirectly inhibiting RET kinase activity, thereby blocking downstream signaling pathways and suppressing tumor growth. Clinical trials have demonstrated excellent efficacy, and some of these drugs have received FDA approval for treating KIF5B-RET-positive lung cancer^[114]^.

Beyond selective RET inhibition, research has increasingly focused on developing dual RET/ALK inhibitors, which may provide clinical advantages in tumors with complex resistance mechanisms or concurrent oncogenic alterations. Notably, Song *et al.* identified benzo[b]kazolone, a compound initially designed as an ALK inhibitor, which also exhibits potent activity against both wild-type and mutant forms of ALK (including L1196M and C1156Y), as well as mutant RET^[115]^. This compound demonstrated low nanomolar IC_50_ values across multiple kinase targets, indicating a broad-spectrum inhibition profile. *In vivo* xenograft studies further confirmed its robust antitumor efficacy, supporting its potential application in NSCLC cases driven by RET fusions or those with acquired ALK inhibitor resistance. These findings suggest that dual RET/ALK inhibition may represent a promising strategy to overcome limitations of single-agent kinase inhibitors^[116]^.

It is important to note that KIF5B-RET targeted therapy is not suitable for all lung cancer patients; genetic testing is necessary to identify candidates. Additionally, targeted therapies may cause side effects and can still encounter resistance, requiring careful evaluation and management by healthcare providers^[117]^. In summary, KIF5B-RET targeted therapy offers a novel approach that offers renewed hope for patients with ALK-targeted therapy-resistant lung cancer. Accurate molecular subtyping and target screening are critical to enabling personalized treatment strategies for patients with acquired resistance.

### Immunotherapy

**Figure 4:**
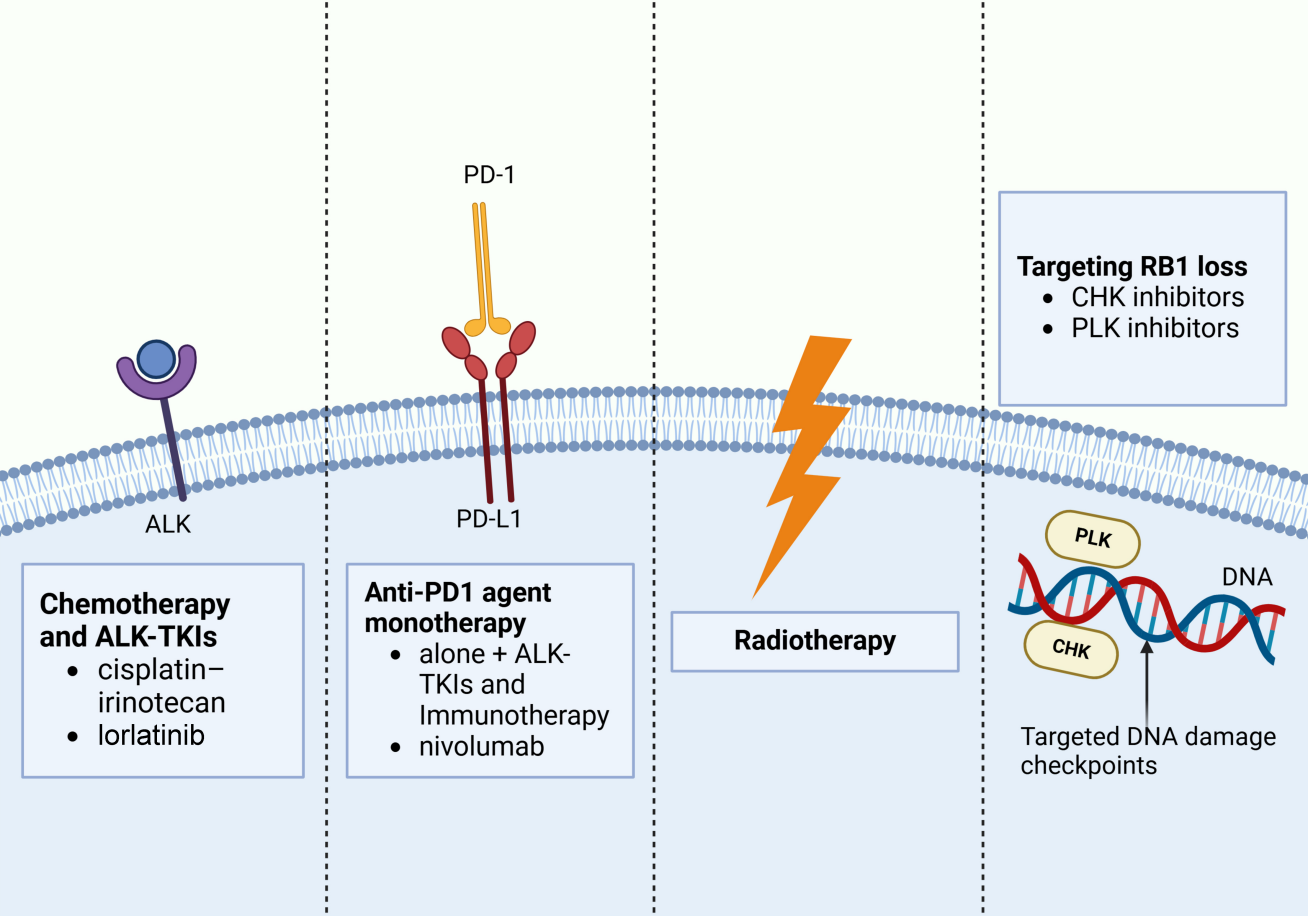
Potential therapeutic targets for transformed SCLC from ALK-mutant NSCLC. These treatments include chemotherapy, ALK-TKIs, immunotherapy, radiation therapy, and CHK and PLK inhibitors targeting RB1 loss. Created in BioRender. Long, M. (2025). https://BioRender.com/gtgq662. SCLC: Small cell lung cancer; ALK: anaplastic lymphoma kinase; NSCLC: non-small cell lung cancer; TKIs: tyrosine kinase inhibitors; CHK: checkpoint kinase; PLK: polo-like kinase.

Immunotherapy activates the patient’s immune system to recognize and eliminate cancer cells. Although it is not yet widely established for the treatment of ALK-targeted therapy-resistant lung cancer, some studies suggest that immunotherapy may offer benefits in ALK-positive NSCLC^[118,119]^. In recent years, ICIs (such as PD-1 and PD-L1 inhibitors) have shown efficacy in lung cancer treatment, including ALK-positive cases^[120,121]^ [Figure 4]. Evidence also indicates that ALK inhibitors can enhance the effects of immunotherapy, making combination regimens a potential strategy for patients with ALK-targeted therapy resistance. Several clinical trials are currently exploring this approach. The Phase III KEYNOTE-789 trial (NCT03515837) is evaluating chemotherapy with or without Pembrolizumab in NSCLC patients, while the Phase III ATLAS trial (NCT03991403) is assessing the combination of atezolizumab and bevacizumab with chemotherapy in patients harboring EGFR mutations or ALK rearrangements^[29]^. Crizotinib, a first-line ALK inhibitor for advanced ALK-positive NSCLC, demonstrates initial efficacy; however, disease progression is inevitable. In contrast, immunotherapy approaches, including PD-1 inhibitors such as Nivolumab, have produced durable clinical responses and contributed to prolonged OS in some lung cancer patients^[85]^. Notably, results from the ALK/EGFR-positive subgroup of the IMpower150 trial demonstrated that combination therapy significantly improved patient survival, suggesting that this approach may partially overcome the immune tolerance associated with ALK-positive tumors^[122]^. Moreover, novel PD-1/VEGF bispecific antibodies, such as AK112, have shown promising efficacy in ongoing clinical trials, offering new perspectives for future combination strategies^[123]^. Currently, several ICIs, including Nivolumab, Atezolizumab, Durvalumab, and Pembrolizumab, have received regulatory approval for the treatment of lung cancer. The introduction of these agents has transformed the therapeutic landscape of advanced NSCLC, markedly improving outcomes in certain patient populations^[124]^. Although ICI monotherapy has shown limited efficacy in ALK-positive NSCLC, combining ICIs with chemotherapy, anti-angiogenic therapy, or second-/third-generation ALK-TKIs may represent a promising direction to overcome therapeutic resistance and provide renewed hope for affected patients.

### Multitarget therapy

In addition to *ALK* gene fusions, ALK-positive lung cancer may also harbor other molecular alterations that contribute to tumor growth and progression. Therefore, simultaneously targeting multiple molecules may be more effective in controlling disease progression in cases with acquired resistance to ALK-targeted therapy. Recent evidence suggests that dual targeting of ALK and EGFR or HER2 can provide therapeutic benefits in ALK inhibitor-resistant lung cancer^[125,126]^. For example, combining ALK inhibitors with EGFR inhibitors (such as Gefitinib) suppresses both ALK and EGFR signaling pathways^[127,128]^. In transgenic mouse models carrying EGFR mutations (Del19-T790M and L858R-T790M), the combination of Crizotinib and the third-generation EGFR inhibitor WZ4002 exhibited potent antitumor activity^[129]^. Similarly, in NCI-H3122 cells with hyperactivated EGFR signaling, resistance to Ceritinib and Alectinib was effectively overcome by co-treatment with the EGFR inhibitor Afatinib^[130]^. Osimertinib, a third-generation EGFR TKI that irreversibly binds its target, has been established as the standard first-line treatment for NSCLC patients with EGFR mutations. In the FLAURA trial, Osimertinib achieved superior OS and progression-free survival (PFS) compared with first-generation EGFR TKIs such as Erlotinib and Gefitinib. The subsequent FLAURA-2 study further indicated that combining chemotherapy with Osimertinib extended PFS from 16.7 to 25.5 months, highlighting the potential synergistic benefits of multitargeted strategies in specific oncogenic contexts^[131,132]^.

Several novel multitarget TKIs have also demonstrated promising clinical potential. Entrectinib, a broad-spectrum inhibitor of tropomyosin receptor kinase (TRK), ROS1, and ALK, has shown efficacy in tumors with diverse gene fusions and is under evaluation in the Phase II STARTRK-2 clinical trial^[133]^. Iruplinalkib (WX-0593) and Envonalkib (TQ-B3139), which selectively target ROS1, ALK, and MET, have received approval in China to treat locally advanced or metastatic ALK-positive NSCLC in patients who progressed on or were intolerant to Crizotinib^[134,135]^. Collectively, these findings suggest that multitargeted approaches may help overcome resistance to ALK inhibitors and improve clinical outcomes. Systematic evaluation of resistance mechanisms and dynamic adjustment of therapeutic regimens will be essential to optimize long-term survival.

### Novel ALK-targeted drugs

Several novel ALK-targeted drugs have been developed and are currently being tested in clinical studies. These next-generation inhibitors suppress ALK protein activation through different mechanisms, thereby helping to overcome resistance to earlier ALK inhibitors. For instance, Lorlatinib, a third-generation ALK inhibitor approved by the FDA for the treatment of ALK-positive NSCLC, effectively inhibits a wide range of ALK variants, including those that have acquired resistance to prior ALK-TKIs^[136]^. It has demonstrated strong activity against multiple acquired ALK mutations, such as G1202R, and can restore sensitivity in patients resistant to Crizotinib. Lorlatinib also shows potent inhibitory activity against ALK and ROS1 mutations and resistance mechanisms^[137,138]^. However, with continued use, new compound mutations may arise, ultimately leading to resistance to Lorlatinib itself.

To address drug-resistant mutations caused by long-term ALK-TKI therapy, fourth-generation ALK inhibitors such as NVL-655 (Neladalkib) and TPX-0131 have been created. These agents were designed to exhibit “dual-mutation activity”, allowing them to simultaneously inhibit two co-occurring mutations within the ALK kinase domain^[133]^. TPX-0131 demonstrates strong central nervous system (CNS) penetration and binds tightly to the adenine region of the ATP-binding site of ALK kinase, thereby demonstrating strong activation. In preclinical studies, it showed potent inhibitory effects against a variety of ALK resistance mutations, particularly G1202R single mutations and L1196M/G1202R compound mutations^[55]^. TPX-0131 is over 100-fold more active against G1202R than Lorlatinib, though its activity against I1171N and G1269S is comparatively limited. In animal models, TPX-0131 achieved complete tumor regression and reached cerebrospinal fluid concentrations of up to 66% of plasma levels, confirming robust CNS penetration^[139]^. Despite these promising results, TPX-0131 was withdrawn from the Phase I trial (NCT04849273) due to an unsatisfactory risk-benefit profile. In contrast, NVL-655 is advancing more successfully in clinical development. It was designed to improve ALK selectivity while reducing off-target inhibition of the TRK family, which is expected to minimize CNS toxicity. NVL-655 exhibits potent inhibitory activity against multiple single and compound ALK resistance mutations, including G1202R, I1171N, G1202R/L1198F, and G1202R/L1196M, among others^[140]^. Importantly, its very low inhibitory activity against TrkB suggests a reduced risk of neurotoxicity. In mouse tumor models based on the EML4–ALK V1 fusion gene, NVL-655 demonstrated superior antitumor efficacy compared with Lorlatinib. The ongoing Phase I/II clinical trial ALKOVE-1 (NCT05384626) has reported favorable CNS penetration and manageable toxicity and side effects in heavily pretreated patients with ALK-positive NSCLC^[141]^. Phase I focused on dose escalation and safety, while Phase II is evaluating the objective response rate (ORR) as assessed by an independent review committee.

Beyond TPX-0131 and NVL-655, other novel ALK inhibitors, such as ZX-29, Gilteritinib, and dual-target compounds like CHMFL-ALK/EGFR-050 and CEP-37440, have shown promising preclinical efficacy. These agents are effective against diverse ALK resistance variants and may offer new therapeutic avenues, particularly for patients with compound mutations or brain metastases^[142]^. Nonetheless, these investigational drugs remain at an early stage of clinical development, and further studies are required to validate their efficacy and safety. Careful evaluation of potential side effects and long-term safety profiles will also be essential.

### Combination therapy

Building on ALK-targeted drug therapy, additional treatment approaches such as chemotherapy, radiotherapy, and immunotherapy are increasingly being incorporated. With advances in understanding the mechanisms of lung cancer drug resistance and in molecular biology technologies, the treatment of NSCLC has become progressively more complex and diverse^[143]^. Developing treatment plans based on genetic information is essential for selecting the most appropriate strategies, both for ALK-TKIs and for combination regimens following the emergence of resistance^[144]^. Multidisciplinary collaboration and the accumulation of clinical expertise are also required to manage adverse events effectively^[145]^. In targeted therapy for lung cancer, it is essential to realize that tumor heterogeneity is dynamic, with ongoing changes in cancer-driving genes and epigenetic modifications. Therefore, repeated biopsies and comprehensive analyses of tumor cells and their microenvironment are particularly important to dynamically monitor the factors driving tumor progression.

## CONCLUSION AND PROSPECT

Lung cancer is characterized by high morbidity and mortality and is prone to recurrence and metastasis, making early diagnosis and prognostic markers critically important. In epigenetic research, the identification of DNA hypermethylation patterns in transcription factor gene promoter sequences has revealed homeobox (HOX)-related genes that may serve as biomarkers for early detection and prognostic evaluation of lung cancer. In addition, dysregulated long non-coding RNAs (lncRNAs), such as HotTip, BLACAT1, and SOX2/ANRIL, have been detected in the serum of NSCLC patients. These circulating lncRNAs show potential as early diagnostic markers and may be associated with OS. Continued exploration of epigenetic mechanisms is likely to yield additional biomarkers, providing valuable guidance for improving early detection and prognostic assessment of NSCLC. Targeted therapy for ALK-positive NSCLC has significantly improved patient prognosis. However, the development of drug resistance has become a major clinical challenge. Current strategies to address resistance include performing re-biopsies to identify underlying mechanisms, adjusting treatment regimens, and investigating novel approaches such as combination targeted therapy and immunotherapy. Although second- and third-generation ALK-TKIs have achieved notable clinical success, resistance remains an unresolved problem.

In summary, resistance to ALK-targeted therapy in NSCLC is inevitable and multifactorial. Resistance mechanisms are highly heterogeneous and include ALK kinase domain mutations, bypass signaling activation, histological transformation, and immune escape. Recent studies have also highlighted the influence of the tumor immune microenvironment and checkpoint molecule regulation (e.g., PD-L1 upregulation) on resistance. Moreover, small cell transformation has emerged as a distinct and aggressive resistance phenotype that requires early detection and individualized management. Based on current knowledge, a comprehensive strategy that integrates dynamic monitoring of resistance mutations, molecular re-biopsy, and rational combination therapy (including next-generation ALK inhibitors, immunotherapy, or dual-target agents) is essential to improve long-term outcomes. Future research should aim to combine molecular profiling with immune landscape characterization to guide truly personalized treatment for ALK-positive NSCLC.

Overall, a deeper investigation into the molecular mechanisms of drug resistance and the immune microenvironment in ALK-positive NSCLC is crucial for designing more effective therapeutic strategies. Future efforts should focus not only on optimizing diagnosis and treatment at early stages but also on developing comprehensive treatment strategies that combine effective drugs with rational sequencing. As more next-generation drugs gain regulatory approval, their optimal clinical application must be carefully defined. At the same time, supportive care and toxicity management remain indispensable for maximizing treatment benefits and survival. Thus, future research should advance both fundamental understanding and clinical practice to achieve more precise and personalized therapies for patients with ALK-positive NSCLC.

## DECLARATIONS

### Copyright

© The Author(s) 2025.
